# OCCUPATIONAL PERFORMANCE ONE TO FIVE YEARS AFTER ANEURYSMAL SUBARACHNOID HAEMORRHAGE: A COHORT STUDY

**DOI:** 10.2340/jrm.v56.24187

**Published:** 2024-03-20

**Authors:** Marcus KESSNER, Jan MEHRHOLZ, Svein Harald MØRKVE, Tina TAULE

**Affiliations:** 1 Department of Occupational Therapy, Haukeland University Hospital, Bergen, Norway; 2 Department of Public Health, Dresden Medical School, Technical University Dresden, Germany; 3 Department of Neurosurgery, Haukeland University Hospital, Bergen, Norway; 4 Bachelor in Occupational Therapy, Faculty of Health and Function, Western Norway University of Applied Sciences, Bergen, Norway

**Keywords:** activities of daily living, intracranial haemorrhages, social participation, patient-reported outcome measures

## Abstract

**Objective:**

To report on the self-perceived occupational performance of patients with aneurysmal subarachnoid haemorrhage and examine the associations between aneurysmal subarachnoid haemorrhage characteristics, socio-demographic factors and self-perceived problems.

**Design:**

A single-centre cohort study design was combined with a cross-sectional analysis.

**Subjects/patients:**

All patients with aneurysmal subarachnoid haemorrhage who were capable of performing activities of daily living before discharge from hospital were included.

**Methods:**

The assessment of the patient’s occupational performance followed a patient-reported outcome measure 1 to 5 years after the subarachnoid haemorrhage. Secondary outcomes comprised scores from the Glasgow Outcome Scale, modified Rankin Scale, Fisher Scale, World Federation of Neurological Societies grading system, vasospasm, and hydrocephalus.

**Results:**

Of the 62 patients included in the study (66% female, mean age 55 years), 79% reported experiencing issues with occupational performance, most frequently with regard to leisure and productivity. The problems reported were significantly associated with vasospasm (*p* = 0.021) and the Glasgow Outcome Scale score (*p* = 0.045).

**Conclusion:**

Even patients who have had aneurysmal subarachnoid haemorrhage with a favourable outcome may encounter occupational performance difficulties for several years. It is vital to use patient-reported outcome measures to identify these issues. This research enhances our comprehension of aneurysmal subarachnoid haemorrhage patients’ self-perceived occupational performance and the factors that affect their performance.

LAY ABSTRACT

This study focused on patients with aneurysmal subarachnoid haemorrhage who were able to perform daily activities independently before being discharged from hospital. We studied how these patients felt about their occupational performance 1 to 5 years after their haemorrhage. We also looked at how socio-demographic factors and aneurysmal subarachnoid haemorrhage characteristics impacted their perceived problems. Of the 62 patients examined, 79% reported facing 1 or more difficulties with activities of daily living, especially in leisure and productivity. The individuals who had documented moderate disability or recorded vasospasm while hospitalized had a notably higher frequency of occupational performance problems than others. The consequences of aneurysmal subarachnoid haemorrhage are felt by many patients for years after the onset of the disease, as our findings show. This study helps to understand the circumstances of aneurysmal subarachnoid haemorrhage patients.

Aneurysmal subarachnoid haemorrhage (aSAH) is a type of stroke caused by a ruptured brain aneurysm (1). The global incidence of aSAH is approximately 6.7 per 100,000 persons (2) and is slightly more common in women than in men (3). This kind of haemorrhage mainly affects people of working age at an average age of 55 years (4). Due to the young age of those affected, aSAH results in the loss of many years of productive life, which affects not only the individual but also society (5). The likelihood of a patient surviving aSAH has increased remarkably over the last decades and is now around 65% (6); only one-third of survivors are documented as having a favourable outcome on discharge from hospital (7). The Glasgow Outcome Scale (GOS) (8), a commonly used clinician-reported outcome measure, indicates a favourable outcome when the patient is able to independently perform daily activities without assistance (9). However, the GOS is not specifically designed for patients with aSAH (10) and it is reported that a clinician-reported outcome measure, such as the GOS, may not be sensitive enough to detect all aspects of the patient’s recovery and independence in daily activities (11). For patients with favourable results, the management at and after discharge from hospital is not yet standardised across national borders or between hospitals (7). However, it is common practice to discharge these patients to their home environment as soon as radiological imaging shows structural normality and generic measures indicate independence in daily activities (7).

An important question is whether these patients with a favourable outcome are capable of managing their occupations and their daily activities, whether immediately after their hospitalisation or in the following years (12, 13). Several studies have shown that many aSAH patients with a favourable outcome are unable to return to their pre-morbid functional level even after several years (14–16). Performing daily activities usually requires both decision-making and problem-solving skills, skills that aSAH patients are frequently well documented as struggling with (5, 16). Al-Khindi et al. (17) state in their review that the haemorrhage affects day-to-day functioning, but that it is necessary to learn more about the long-term effects of aSAH on complex daily activities related to the ability to live independently in the community. To our knowledge, previous studies focusing on aSAH patients with favourable outcomes do not identify self-perceived problems of occupational performance. There is also limited information on whether and to what extent aSAH characteristics or demographic factors influence occupational performance (7, 18, 19).

Given the lack of information concerning the occupational performance of aSAH patients with a favourable outcome, the aim of this study is twofold: (1) to describe patients’ self-perceived occupational performance in self-care, productivity and leisure in the period 1 to 5 years after aSAH; and (2) to investigate the association between aSAH characteristics and socio-demographic factors with self-reported occupational performance problems.

## METHODS

### Study design

**Fig. 1:**
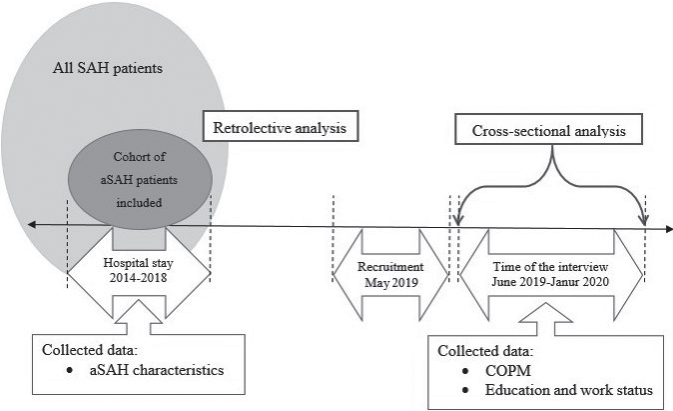
Illustration of the study designs. aSAH: aneurysmal subarachnoid haemorrhage; COPM: Canadian Occupational Performance Measure; SAH: subarachnoid haemorrhage.

The present cohort study combines a retrolective design (20) with a cross-sectional analysis (21). Fig. 1 illustrates the study design. This combination of two designs made it possible to examine the clinical status of the participants descriptively at the time of their hospitalisation and then further analyse the associations with the participants’ current life situation (20, 21).

### Participants

Patients with an aSAH, as indicated by code I60 of the International Statistical Classification of Diseases and Related Health Problems (ICD-10), were eligible to participate if they had been treated at the Department of Neurosurgery at Haukeland University Hospital (HUH) in Bergen, Norway between January 2014 and December 2018. To satisfy inclusion criteria, patients needed to be over 18 years of age at the time of recruitment, have a good level of recovery indicated by a GOS score of ≥4 at the time of discharge from hospital, and be able to perform daily activities independently or mostly independently as indicated by a modified Rankin Scale (mRS) score of ≤3 at the time of discharge from hospital. Patients who did not speak or understand Norwegian were excluded from the study, as were patients with severe language problems or insufficient mental capacity to participate in a telephone interview.

### Data collection

The outcome measures and time of data collection are given in Table I. The primary outcome was patients’ self-perceived occupational performance as measured by the Norwegian version of the Canadian Occupational Performance Measure (COPM) (22). The secondary outcome variables were the aSAH characteristics, which were collected from the patients’ journals. The background variables were information regarding the participants’ age, gender, level of education and work status, collected by way of a custom-designed questionnaire. To obtain a comprehensive understanding of the severity of the haemorrhage the Fisher Scale (23), the World Federation of Neurological Societies (WFNS) grading system (24), and complication vasospasm (VS), complication hydrocephalus (HS) as well as the GOS (8) were recorded. To describe the degree of recovery the mRS (10) were used. Both the COPM and the custom-designed questionnaire were assessed during an interview in the follow-up in the period 1 to 5 years after the aSAH. The first author conducted all interviews. Each interview lasted between 20 and 45 minutes.

**Table I:** Overview of the chronological process of the study, with the time point of data collection for the primary and secondary outcome and the enrolment

Study period	January 2014–December 2018		May 2019	June 2019–January 2020
	Hospital admission	Discharge from hospital	First patient contact	Interview
Recruitment				
Informed consent			X	
Primary outcome measure				
Canadian Occupational Performance Measure				X
Secondary outcome measures				
Age	X			
Gender	X			
Education and work status				X
Background variables				
World Federation of Neurological Societies	X			
Fisher scale	X			
Modified Rankin Scale		X		
Glasgow Outcome Scale		X		
Complications		X		

*Canadian Occupational Performance Measure*. The COPM is a patient-reported outcome measure that is validated for use in clinical practice (22, 25). In this study, the COPM was conducted by telephone. The telephone interview enabled participants from a large geographical area to be included in the study. A study by Korner-Bitensky et al. has shown that data collected from a telephone interview are comparable to data collected from a face-to-face interview (26), thus justifying this approach.

The COPM was performed in four steps as described by Kjeken et al. (22). In step 1, the participants were asked to describe the occupations they considered to be important but difficult to perform due to the aSAH. In step 2, the participants rated the importance (COPM-I) of each difficult occupation identified in step 1. A visual analogue scale (VAS) ranging from 1 to 10 was used. Higher scores indicated greater importance. In step 3, patients rated both their performance (COPM-P) and their satisfaction (COPM-S) with their performance for each of the difficult occupations identified in step 1. On a VAS of 1 to 10, the participants rated their COPM-P score, where 1 = not able to perform and 10 = able to perform extremely well. They then rated their COPM-S score on a VAS of 1 to 10, where 1 = not satisfied at all and 10 = extremely satisfied. In step 4, the average COPM-P score and COPM-S score were calculated.

*The custom-designed questionnaire*. In the custom-designed questionnaire, the patients’ level of education was documented by level: Level 1 – elementary school; Level 2 – high school; Level 3 – university (27). To document the participants’ work situation and possible changes to it, it was recorded at 3 different points in time: before the aSAH, when the participants returned to work and at the time of the aSAH. The length of time between the aSAH and the return to work was also recorded. In addition, the current living situation in work terms was documented according to whether the participants were working, retired, looking for work, on leave from work, receiving disability benefits or in another living situation.

*Fisher scale*. The Fisher scale is a classification of the amount of subarachnoid haemorrhage on computed tomography (CT) scans and thus indicates the severity of the aSAH (23). Patients are categorised into 4 different groups according to the extent of bleeding: Grade 1 – no bleeding is discernible; Grade 2 – the bleeding is less than 1 mm in depth; Grade 3 – the bleeding is 1 mm or greater in depth; Grade 4 – bleeding with intraventricular haemorrhage or parenchymal extension (23).

*World Federation of Neurological Societies grading system*. The WFNS grade also indicates the severity of the aSAH but in clinical terms (24). The WFNS grading system evaluates the neurological condition of the patient after admittance, which it assesses using both the Glasgow Coma Score (GCS) (28) and focal neurological deficits. WFNS grade 1 indicates a GCS of 15 and no motor deficit. At WFNS grade 2, the patient is classified as having a GCS of 13 to 14 and no focal neurological deficit. At WFNS grade 3, the patient also has a GCS of 13 to 14 but has an additional focal neurological deficit. A WFNS grade 4, the patient has a GCS of 7 to 12 and may or may not have a neurological deficit. At WFNS grade 5, the patient has a GCS of 3 to 6 and may or may not have a neurological deficit (24).

*Glasgow Outcome Scale*. The GOS is used to classify outcomes for patients according to the severity of brain damage (8). The patient’s physical and neurological status, as well as their neuropsychological condition, are used to describe their degree of recovery in a standardised and objective manner. GOS scores are defined as follows: Grade 1 – death; Grade 2 – the patient reaches a persistent vegetative state; Grade 3 – the patient has severe disability due to a mental or physical disability, or a combination of both; Grade 4 – moderate disability, no need for assistance in everyday life, employment is possible but may require special equipment; and Grade 5 – a good recovery and resumption of normal life despite minor deficits (8). With regard to patients with traumatic brain injury, the result of the GOS has often been dichotomised as unfavourable outcome for grades 0 to 3 and favourable outcome for grades 4 and 5. A literature review shows that the GOS is not specifically designed for aSAH patients and may therefore have limitations when measuring patients’ degree of recovery (11).

*Modified Rankin Scale*. The mRS is a clinician-reported scale for degree of disability or the dependency of people who have suffered a stroke or other neurological disease (10). The scale ranges from 0 to 5. High scores mean a higher degree of disability (10, 29). Specifically, mRS grade 0 means the patient has no symptoms. At grade 1, the patient has no significant disability and is able to perform all normal activities despite some symptoms. Grade 2 indicates a slight disability. The patient is able to look after his or her own affairs without assistance but is unable to carry out all previous activities. Grade 3 indicates a moderate disability. The patient requires some help but is able to walk unassisted. At grade 4, the patient has a moderately severe disability. The patient is unable to attend to his or her own bodily needs without assistance and is unable to walk unassisted. In the case of a severe disability, where the patient requires constant nursing care and attention and is bedridden and incontinent, the patient is graded at 5. An additional category, death (grade 6), is usually included in clinical studies.

The mRS is a widely used clinical measurement tool in modern stroke studies with documented validity and reliability in term of assessing the impact of new stroke treatments (29). However, when applied to aSAH, the mRS has well-defined limitations (11).

### Sample size estimation

The estimated number of participants is based on the 5-year report for 2008–2012 of the Neurosurgery Unit of HUH (30). This report shows that 237 patients underwent acute aSAH treatment in the years 2008 to 2012. Of these patients, 142 achieved a GOS score of > 4, indicating a favourable outcome (9). On the basis of this calculation, we expected about 28 patients per year to be enrolled in our study. Calculated over a period of 5 years, the total number of potential participants was expected to be approximately 125.

### Statistical analyses

SPSS 24.0 (IBM Corp, Armonk, NY, USA) was used for statistical analysis. To examine whether the study’s group of participants differs from the group of the non-participants, mean value comparisons were carried out by way of the exact χ^2^ test for categorical variables, the *t*-test for continuous variables, and the Mann–Whitney *U* test for continuous but not normally distributed variables. A mean value comparison with the Kruskal–Wallis was used to compare the ordinal scale level with the numerical scale level. The Mann–Whitney *U* test was performed to compare the categorical variable with the numerical scale level. Variables that had a *p*-value below 0.05 in this analysis were included in an Eta correlation. The significance level for the Eta correlation coefficient was set at a *p*-value below 0.05. No correction for multiple analysis was performed due to the limited number of patients in the study.

## RESULTS

### Participants

**Fig. 2:**
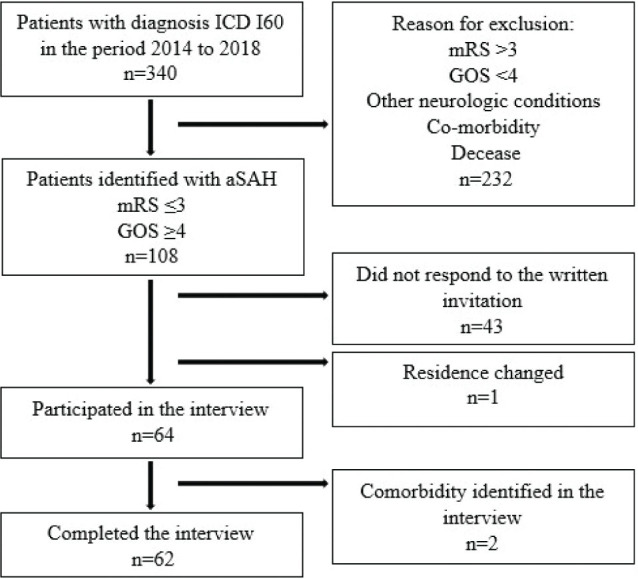
Flow diagram depicting the inclusion sequence and the reasons for exclusion. aSAH: aneurysmal subarachnoid haemorrhage; ICD: International Statistical Classification of Diseases and Related Health Problems code; mRS: modified Rankin Scale; GOS: Glasgow Outcome Score.

Fig. 2 illustrates the inclusion and exclusion process. Between January 2014 and December 2018, 340 patients suffered aSAH and were admitted to the HUH in Bergen, Norway. Of these, 108 patients met the inclusion criteria and 64 agreed to participate in the study. Two patients were excluded because they were not able to clearly relate their occupational performance problems to the aSAH due to comorbidities. A total of 62 participants were included in the study.

The participants’ socio-demographic characteristics and their state of employment before the aSAH, at the time they resumed work and at the time of the interview are all listed in Table II.

**Table II:** Socio-demographic characteristics and the state of employment of included participants

Socio-demographic variables	Participants *n* = 62
Gender, female, *n* (%)	41 (66)
Age at aSAH in years, mean (SD)	55 (10)
Time from aSAH to interview in years, mean (SD)	3 (1)
Education, *n* (%)	
Elementary school	4 (7)
High school	31 (50)
University	27 (43)
Time from aSAH to work resumption in months, mean (SD)	6 (6)
Employment status, *n* (%)	
T1	
Working	55 (89)
Not working	7 (11)
T2	
Working	47 (76)
Not working	15 (24)
T3	
Working	34 (55)
Not working	28 (45)
Living situation according to work at the time of the interview, *n* (%)	
Working	34 (55)
Retired	10 (16)
Seeking employment	1 (2)
Disability benefits	13 (21)
Other	4 (7)

aSAH: aneurysmal subarachnoid haemorrhage; SD: standard deviation; T1: time point before bleeding event; T2: time point at work resumption; T3: time point of the interview.

Of the 108 patients who met the inclusion criteria, those in the group of participants did not differ significantly from those in the group of non-participants with regard to sociodemographic variables and aSAH characteristics. Mean comparisons of all documented variables are presented in Table III.

**Table III:** Socio-demographic variables and aSAH characteristics of participants and non-participants and a mean value comparison of these two groups

Variables	Total *n* = 108	Non-participants *n* = 46	Participants *n* = 62	Differences between groups *p*-value
Socio-demographic variables				
Gender, *n* (%)				
Female	65 (60)	24 (52)	41 (66)	
Male	43 (40)	22 (48)	21 (34)	0.143^a^
Age at aSAH in years, mean (SD)	53 (11)	52 (11)	55 (10)	0.355^b^
Clinical variables at time point of hospital admission				
WFNS scale, *n* (%)				
1	65 (60)	31 (67)	34 (55)	0.266^b^
2	28 (26)	9 (20)	19 (31)	
3	3 (3)	1 (2)	2 (3)	
4	9 (8)	3 (7)	6 (10)	
5	3 (3)	2 (4)	1 (2)	
Fisher Scale, *n* (%)				
1	9 (8)	1 (2)	8 (13)	0.147^b^
2	21 (19)	9 (20)	12 (19)	
3	54 (50)	24 (52)	30 (48)	
4	24 (22)	12 (26)	12 (19)	
Clinical variables at time point of hospital discharge				
mRS score, *n* (%)				
1	21 (19)	7 (15)	14 (23)	0.352^b^
2	32 (30)	15 (33)	17 (27)	
3	24 (22)	8 (17)	16 (26)	
4	31 (29)	16 (35)	15 (24)	
GOS score, *n* (%)				
4	31 (29)	16 (35)	15 (24)	0.229^a^
5	77 (71)	30 (65)	47 (76)	
HC, *n* (%)				
No	49 (45)	22 (48)	27 (43)	0.659^a^
Yes	59 (55)	24 (52)	35 (57)	
VS, *n* (%)				
No	90 (83)	37 (80)	53 (85)	0.486^a^
Yes	18 (17)	9 (20)	9 (15)	

a Exact χ^2^ test for categorical variables;

b Mann–Whitney *U* test.

aSAH: aneurysmal subarachnoid haemorrhage; GOS: Glasgow Outcome Score; HC: hydrocephalus; mRS: modified Rankin Scale; SD: standard deviation; VS: vasospasm; WFNS: World Federation of Neurosurgical Societies.

### Participants’ self-perceived occupational performance problems after aneurysmal subarachnoid haemorrhage

A total of 49 participants reported at least 1 or more occupational performance problems, with a mean of 4 problems (SD ± 3) related to aSAH. A total of 256 problems were reported by the participants. Participants described their occupational performance problems as often being caused by fatigue, lack of energy, headaches or cognitive deficits such as memory and concentration problems.

Of all the occupational performance problems, 120 were related to the COPM category of leisure. In this category of COPM, participants reported, among other things, reduced social activity due to a lack of energy or headaches that could occur in the company of other people, difficulties in controlling emotions, and specific memory and concentration difficulties that affected their ability to read, watch TV or play board games. In the COPM category of productivity, the participants reported 94 occupational performance problems. For example, participants referred to reduced endurance and concentration, with a need for more frequent breaks, and the experience of reduced memory function. The fewest problems were identified in the category of self-care. The participants reported 41 occupational performance problems related to this category, such as taking more time for personal hygiene, not showering due to lack of energy, limited walking due to vertigo, difficulty climbing stairs and so on.

**Fig. 3:**
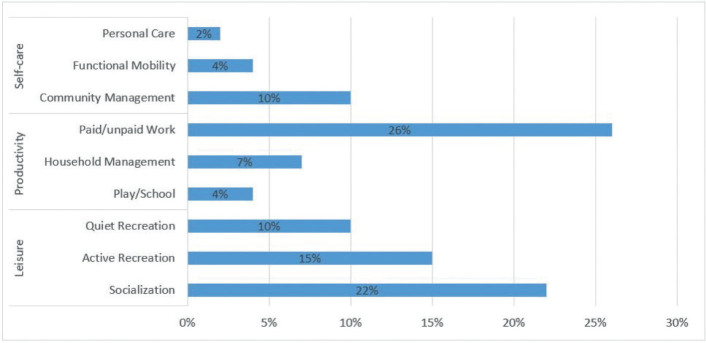
Percentage distribution of all occupational performance problems identified with the Canadian Occupational Performance Measure.

The distribution of the participants’ self-perceived occupational performance problems in all 3 COPM categories are summarised in Fig. 3.

For all identified occupational performance problems, the participants had a mean COPM-I score of 7 (SD ± 2), a mean COPM-P score of 6 (SD ± 2) and a mean COPM-S score of 5 (SD ± 2).

### Associations between socio-demographic factors and aneurysmal subarachnoid haemorrhage characteristics with self-perceived occupational performance problems

Table IV compares aSAH characteristics and socio-demographic factors with mean COPM scores. Participants diagnosed with VS had a statistically significantly higher incidence of occupational performance problems than those not diagnosed with VS. Participants classified as having moderate disability on the basis of the GOS reported significantly more occupational performance problems in the area of self-care than did patients classified as having a good recovery.

**Table IV:** Comparison of aneurysmal subarachnoid haemorrhage (aSAH) characteristics and socio-demographic factors with mean values of COPM scores

Variables	*N*	COPM numbers of problems			
		Total	Self-care	Productivity	Leisure
Clinical variables – aSAH characteristics					
WFNS scale grade, mean (SD)					
1 2 3 4 5	34 19 02 06 01	4 (3) 3 (3) 10 (7) 4 (4) –	1 (1) 0 (1) 4 (4) 1 (1) –	2 (1) 1 (1) 3 (2) 1 (1) –	2 (1) 1 (1) 4 (1) 2 (2) –
*p*-value^a^		0.232	0.050	0.563	0.385
Fisher Scale score, mean (SD)					
1 2 3 4	08 12 30 12	3 (4) 4 (3) 5 (3) 3 (3)	0 (1) 1 (1) 1 (1) 0 (1)	1 (1) 2 (1) 2 (1) 1 (1)	2 (1) 2 (1) 2 (2) 1 (1)
*p*-value^a^		0.237	0.181	0.200	0.409
mRS score, mean (SD)					
0 1 2 3	14 17 16 15	4 (3) 4 (3) 4 (3) 5 (4)	1 (1) 0 (1) 1 (1) 1 (2)	2 (2) 1 (1) 1 (1) 2 (1)	1 (1) 2 (2) 2 (2) 2 (2)
*p*-value^b^		0.551	0.127	0.835	0.241
GOS score, mean (SD)					
4 5	15 47	5 (4) 4 (3)	1 (2) 0 (1)	2 (1) 1 (1)	2 (2) 2 (2)
*p*-value^a^		0.278	0.020*	0.431	0.512
HC, mean (SD)					
No Yes	27 35	3 (3) 5 (3)	0 (1) 1 (1)	1 (1) 2 (1)	2 (2) 2 (2)
*p*-value^a^		0.183	0.078	0.447	0.189
Vasospasm, mean (SD)					
No Yes	53 09	4 (3) 6 (3)	1 (1) 1 (1)	1 (1) 2 (1)	2 (2) 3 (2)
*p*-value^a^		0.016*	0.002*	0.070	0.056
Socio-demographic variables					
Gender, mean (SD)					
Male	21	3 (3)	1 (1)	1 (1)	2 (1)
Female	41	4 (3)	1 (1)	2 (1)	2 (2)
*p*-value^a^		0.290	0.986	0.235	0.224
Education, mean (SD)					
Elementary school	04	3 (4)	1 (1)	1 (2)	2 (1)
High school	31	4 (3)	1 (1)	1 (1)	2 (2)
University	27	4 (3)	1 (1)	2 (1)	2 (2)
*p*-value^b^		0.822	0.099	0.681	0.607

a Mann–Whitney *U* test,

b Kruskal–Wallis test,

* significant *p*-values.

aSAH: ; COPM: Canadian Occupational Performance Measure; GOS: Glasgow Outcome Score; HC: hydrocephalus; mRS: modified Rankin Scale; WFNS: World Federation of Neurosurgical Societies.

No statistically significant associations were found between WFNS grade, Fisher scale score or mRS score and the occurrence of occupational performance problems 1 to 5 years after aSAH.

We performed a correlation analysis to determine whether there was an association between the variables showing statistically a significantly higher incidence of occupational performance problems when compared with the mean values. A summary of all the results is given in Table V. There was a significant positive correlation between the presence of VS and the participants’ total number of self-perceived occupational performance problems, as measured by the COPM. The coefficient of determination indicates that 7% of the total COPM problems described by the participants in this study can be explained by the presence of VS in the acute phase.

**Table V:** Association between occupational performance problems as measured by the COPM 1 to 5 years after the aneurysmal subarachnoid haemorrhage and the independent variables of GOS and VS for all included participants

Variables	COPM – number of problems			
	Total	Self-care	Productivity	Leisure
GOS, *n* = 62				
R	0.174	0.256	0.086	0.095
corrected R^2^	0.014	0.050	–0.009	–0.008
*p*-value^a^	0.177	0.045*	0.509	0.463
VS, *n* = 62				
R	0.292	0.233	0.214	0.249
corrected R^2^	0.070	0.038	0.030	0.047
*p*-value^a^	0.021*	0.069	0.095	0.051

a Eta correlation coefficient; R: correlation coefficient; R^2^: coefficient of determination;

* significant *p*-values.

COPM: Canadian Occupational Performance Measure; GOS: Glasgow Outcome Score; VS: vasospasm.

A significant positive correlation was also documented for the GOS and participants’ self-perceived occupational performance problems in the COPM category of self-care. The statistical calculation indicates that the GOS score can explain 5% of the problems identified in this COPM category.

## DISCUSSION

### Patients’ self-perceived occupational performance problems in leisure, productivity and self-care 1 to 5 years after aneurysmal subarachnoid haemorrhage

The high number of self-perceived occupational performance problems collected with a patient-reported outcome measure, 256 in total, indicates that patients with a favourable outcome after aSAH can also struggle with the consequences of aSAH, even after several years. These findings are similar to those of previous studies, which suggest that many aSAH survivors with a favourable outcome are unable to return to their premorbid functional level (17, 19). In our study, almost half of all identified occupational performance problems relate to leisure activities. This corresponds with previous research in which social functioning was one of the most frequently impaired areas after aSAH (17, 31). In a study by Buunk et al. (32), about half of all patients with aSAH reported an incomplete return to their previous leisure activities and nearly 40% reported changes in social interactions in the chronic phase after suffering aSAH.

The participants in our study who had returned to work reported a considerable number of problems and limitations in their work life, such as functioning at work at the same level as before an aSAH. This is also in line with the results in other studies (5, 16). The study participants had experienced aSAH on average 3 years prior to the interview and returned to work on average 6 months after the haemorrhage. Therefore, it is anticipated that some participants who experienced aSAH less than 3 years ago may face occupational performance issues and may not be able to continue working in the coming years. Additionally, some participants may retire during this period. However, the authors’ perspective suggests that this is only speculation, as the study design does not allow for clear answers. A cross-sectional analysis that follows up on the employment status of all participants 5 years after the bleed would provide a concrete conclusion.

The lowest number of occupational performance problems in our study was documented in the COPM category of self-care. The distribution of occupational performance problems across this COPM category clearly reflects the findings of Tuntland (33), according to which the last function lost is the ability to perform this type of daily activities. However, interpretation of the specific information concerning the kinds of activities aSAH survivors must cope with at different time points clearly requires further examination in future empirical studies.

Another aspect of self-perceived occupational performance as identified by the COPM is participants’ ratings of the importance of each occupation, its performance and their satisfaction with their own performance. The COPM-I scores are an important determinant for identifying a patient’s motivation, cooperation and responsibility in terms of therapeutic interactions (34, 35). The relatively high COPM-I scores in our study suggest that specific therapeutic interventions can be successful in the years following aSAH.

Participants reported that they were able to perform most activities after aSAH but not to the same extent as before the aSAH, and that they were highly dissatisfied with their performance after aSAH. The literature shows that limitations in the performance of daily activities and satisfaction with performance affect quality of life (36, 37). Reduced quality of life has been reported to be associated with aSAH and may persist for several years after a bleeding event (38). In order to broaden the treatment focus for aSAH patients with a favourable outcome, the relationship between occupational performance problems and reduced quality of life should be further investigated.

### Association between aneurysmal subarachnoid haemorrhage characteristics and self-perceived occupational performance problems

This study has found a significant positive but weak correlation between the presence of VS and participants’ perceived occupational performance problems. This finding is comparable to what was reported by Neifert et al. (39), who emphasise that complications such as VS can have a negative impact on patient outcomes. We did not investigate the cause of the possible association we found. Nevertheless, little is currently known about the complication of VS and its impact on functional impairment after aSAH (39).

However, we found a weakly significant positive correlation between the GOS score and self-perceived occupational performance problems. This is in line with the results of Scharbrodt et al., which show that aSAH patients with a GOS grade 5 still had significantly impaired social functioning after several years (40). We also compared the mRS score and self-perceived occupational performance problems. In contrast to the GOS, no significant associations were found. Regardless of the mRS score at the time of hospital discharge, participants described occupational performance problems at the time of the interview. Our findings partially support studies suggesting that half of aSAH patients with an mRS score of 0 have significant cognitive impairment on neuropsychological assessment, with one-third being unable to work (11). Our findings regarding the use of the GOS and the mRS reflect the fact that the use of clinician-reported outcome measures alone is not sufficient for this patient population. The use of patient-reported outcome measures that focus on the performance of daily activities at the time of discharge from hospital should be investigated in further studies.

In the present study, the participants reported occupational performance problems after 1 to 5 years, regardless of the Fisher scale score or the WFNS score. In the review by Al-Khindi, the Fisher scale score was only weakly associated with reduced cognitive function. It was concluded that other factors probably had a bigger impact on cognitive outcome (17). In relation to the WFNS grade, there are mixed results for prognostic characteristics. Some studies suggest that there is a gradual increase in the risk of poor outcome with increasing WFNS grades (14, 41). Other studies have found no correlation between WFNS grade and return to work (42). Further studies are needed to analyse the influence of aSAH severity on occupational performance problems in the years after the bleeding event.

Importantly, effects on occupational performance appear to occur in the years following hospitalisation regardless of the severity of haemorrhage, degree of disability, dependency or HC complication.

### Association between socio-demographic factors and self-perceived occupational performance problems

The overwhelming majority of participants were female, with a mean age of 55 years. This is in line with other studies that have shown the incidence of aSAH to be higher in women than in men, with a mean age of 55 years at presentation (4). In the present study, no associations were found between socio-demographic factors and self-perceived occupational problems. These results suggest that aSAH patients are at high risk of experiencing limitations in their daily activities in the years following their haemorrhage, regardless of their age, gender, education or work situation. A study by Ørbo et al. shows that demographic variables such as age and education level are not predictors of cognitive impairment after aSAH (43). However, other studies confirm significant associations between demographic factors and adverse long-term outcome (14).

### Methodological considerations/limitations

A strength of our study is that there was only a minimum of missing data. Both in the retrolective data analysis and the interviews, complete data sets were collected and evaluated in the statistical analysis.

Another strength of this study is the long follow-up period of 1 to 5 years. A minimum of 1 year means that the effect of spontaneous recovery on the results is minimised. Furthermore, by the time 5 years have passed, patients are no longer supported by the healthcare system. By comparing the baseline characteristics of responders and non-responders and assessing the differences between these groups, confounding factors were to a large extent eliminated. However, some limitations should be considered when making generalisations and drawing conclusions from the results of this study. Only self-reported information was used to assess post-aSAH occupational performance, which could lead to recall bias. The actual performance of the participants’ daily occupations may also not have been accurately reflected (44). In addition, there may be bias due to individual personality traits of the participants. However, subjective measurements are an important and useful supplement for revealing outcomes that are important to aSAH patients (44). In addition, the power of statistical tests may be limited due to the large number of independent variables. Another limitation is the small sample size, which is due to the low prevalence of aSAH patients (2). The results should therefore be interpreted with caution.

### Conclusion

This study focuses on a group of patients with brain haemorrhage and high mortality. The included participants were survivors with a documented favourable outcome at the time of their primary hospitalisation. The overwhelming majority of participants, however, still reported problems with occupational performance in all areas of daily life within the first 5 years of disease onset, regardless of their levels of disability, dependency or aSAH severity. It was found that patients affected by vasospasm or those who reported incomplete recovery may have an increased risk of occupational performance problems.

Despite the limitations, our study makes an important contribution by illustrating the presence of and associations with occupational performance problems in aSAH patients with a favourable outcome. Recovery after aSAH should be assessed in the context of the performance of daily activities and function at home and in society, even after several years. One way to improve the situation of aSAH patients at home is to implement patient-reported outcome measures to assess which important daily activities are limited. If such problems are identified, then interventions and treatments should focus on improving performance and the patient’s own satisfaction with their performance of important daily activities. Helping this group of patients to manage their daily activities may improve their quality of life and limit the socio-economic impact of aSAH.
